# Acid Resisting Mackintoshes

**Published:** 1911-09-16

**Authors:** 

**Affiliations:** Anderson and Anderson, Ltd. 37 Queen Victoria Street, London, E.C.


### INSTITUTIONAL LETTER-BOX.

### ACID RESISTING MACKINTOSHES.

*To the Editor of* THE HOSPITAL.

SIR,—Replying to A. C. M.'s inquiry re acid-proof sheeting, we shall be pleased to forward samples and prices of the above on receipt of his name and address.

Thanking you, we are, Sir, yours faithfully,

ANDERSON, ANDERSON AND ANDERSON, LTD.

37 Queen Victoria Street, London, E.C.

---

MESSRS. W. S. ROTHBAND AND CO., of Bent Street, Manchester, write to us as follows in connection with the same subject :—

We beg to bring before your notice our waterproof bed sheeting, which is made 36 in., 45 in., and 54 in. wide, and of which we herewith attach a sample.

This bed sheeting (which is waterproof and not indiarubber) has been specially prepared for tropical climates, and can be sterilised without its quality or appearance being affected.

It can also be disinfected in 5 per cent. and 10 per cent. carbolic, turpentine, boiling soap and water, chloroform, strong cold ammonia, etc., and still retain its wearing powers, thus offering an immense advantage over the ordinary indiarubber sheeting. Other advantages to be derived from the use of Rothband's sheeting are that it is free from the objectionable odours common to rubber sheetings, and also it can be kept in stock any length of time without deteriorating. It has been tested by immersion in urine for eighteen hours without the slightest alteration.

Another of the many important properties of this bed sheeting is that it can be washed with ordinary soap and water, put away, and it will not get sticky nor crack, whereas the ordinary indiarubber sheetings have all a tendency to get claggy and crack when soap and water are applied. Care must be taken after washing and disinfecting that the sheeting is allowed to dry naturally before folding. Soft soap should not be used.

The price for 36 in. wide is 4s. 6d. per yard net, delivered Manchester. The price for 45 in. wide is 5s. 8d. per yard net, delivered Manchester. The price for 54 in. wide is 6s. 9d. per yard net, delivered Manchester.

A trial is respectfully solicited in order to satisfy you that its working qualities are as we state.

We also make in three grades single-faced sheetings, 36 in. wide, which stand the same tests (on the proofed surface). The prices of these are 1s. 9d., 2s. 3d., and 3s. per yard net. These single-faced sheetings are suitable for accouchement purposes, and are generally stocked by drapers, chemists &c. Samples on application.

We wish to point out that owing to the success of these sheetings on the home market imitations are being made, therefore we perforate every yard on the selvedge W. S. R. M., and without this perforation they are not genuine.

We will be pleased to supply direct or through a merchant.

[We shall be glad to give a report on these sheetings if A. C. M. will favour us with his findings when he has used them.—ED. THE HOSPITAL.]

